# Assessment of Genetic Variability and Evolutionary Relationships of *Rhizoctonia solani* Inherent in Legume Crops

**DOI:** 10.3390/plants12132515

**Published:** 2023-06-30

**Authors:** Aqleem Abbas, Amjad Ali, Azhar Hussain, Amjad Ali, Abdulwahed Fahad Alrefaei, Syed Atif Hasan Naqvi, Muhammad Junaid Rao, Iqra Mubeen, Tahir Farooq, Fatih Ölmez, Faheem Shehzad Baloch

**Affiliations:** 1 Department of Agriculture and Food Technology, Karakoram International University (KIU), Gilgit 15100, Pakistan; dr.amjadali@kiu.edu.pk (A.A.); azhar.hussain@kiu.edu.pk (A.H.); 2 Department of Plant Protection, Faculty of Agricultural Sciences and Technologies, Sivas University of Science and Technology, Sivas 58140, Türkiye; amjadbzu11@gmail.com (A.A.); fatih.olmez@sivas.edu.tr (F.Ö.); 3 Department of Zoology, College of Science, King Saud University, P.O. Box 2455, Riyadh 11451, Saudi Arabia; afrefaei@ksu.edu.sa; 4 Department of Plant Pathology, Faculty of Agricultural Sciences and Technology, Bahauddin Zakariya University, Multan 60800, Pakistan; atifnaqvi@bzu.edu.pk; 5 State Key Laboratory for Conservation and Utilization of Subtropical Agro-Bioresources, Guangxi Key Laboratory of Sugarcane Biology, College of Agriculture, Guangxi University, Nanning 530021, China; 6 State Key Laboratory of Rice Biology, and Ministry of Agriculture Key Laboratory of Molecular Biology of Crop Pathogens and Insects, Institute of Biotechnology, Zhejiang University, Hangzhou 310058, China; iqra.kynat1@gmail.com; 7 Plant Protection Research Institute, Guangdong Academy of Agricultural Science, Guangzhou 510640, China; tfarooq@gdppri.com

**Keywords:** phylogeny, genetic diversity, anastomosis groups, grain legumes, forage legumes, *Rhizoctonia solani*

## Abstract

*Rhizoctonia solani* is one of the most common soil-borne fungal pathogens of legume crops worldwide. We collected rDNA-ITS sequences from NCBI GenBank, and the aim of this study was to examine the genetic diversity and phylogenetic relationships of various *R. solani* anastomosis groups (AGs) that are commonly associated with grain legumes (such as soybean, common bean, pea, peanut, cowpea, and chickpea) and forage legumes (including alfalfa and clover). Soybean is recognized as a host for multiple AGs, with AG-1 and AG-2 being extensively investigated. This is evidenced by the higher representation of sequences associated with these AGs in the NCBI GenBank. Other AGs documented in soybean include AG-4, AG-7, AG-11, AG-5, AG-6, and AG-9. Moreover, AG-4 has been extensively studied concerning its occurrence in chickpea, pea, peanut, and alfalfa. Research on the common bean has been primarily focused on AG-2, AG-4, and AG-1. Similarly, AG-1 has been the subject of extensive investigation in clover and cowpea. Collectively, AG-1, AG-2, and AG-4 have consistently been identified and studied across these diverse legume crops. The phylogenetic analysis of *R. solani* isolates across different legumes indicates that the distinct clades or subclades formed by the isolates correspond to their specific anastomosis groups (AGs) and subgroups, rather than being determined by their host legume crop. Additionally, there is a high degree of sequence similarity among isolates within the same clade or subclade. Principal coordinate analysis (PCoA) further supports this finding, as isolates belonging to the same AGs and/or subgroups cluster together, irrespective of their host legume. Therefore, the observed clustering of *R. solani* AGs and subgroups without a direct association with the host legume crop provides additional support for the concept of AGs in understanding the genetic relationships and evolution of *R. solani*.

## 1. Introduction

The soil-borne fungal phytopathogen *Rhizoctonia solani* Kuhn (teleomorph, *Thanatephorus cucumeris* (Frank) Donk) has a broad host range and is found in various regions across the globe [1,2,3]. This pathogen infects many important crops such as cereals and legumes [4]. Since legumes are crucial for economic development and ecosystem sustainability, they are utilized as major food, feed, and green manure crops [5,6,7,8,9]. Grain legumes, such as soybean, chickpea, pea, cowpea, and beans, are vulnerable to several diseases caused by *R. solani*. These diseases can manifest as blights, including both web and foliar blights, damping-off, and various types of rots affecting different parts of the plant, such as the seed, stem, root, crown, collar, and hypocotyl [10,11,12,13,14,15]. Forage legumes, such as alfalfa and clover, are also susceptible to *R. solani* infections. *R. solani* causes seed rot and damping-off in alfalfa. Similarly, in clover, *R. solani* leads to blight and sheath rot, while in cowpea and peanut, it causes root and stem rot [16,17].

Farmers still rely on chemical methods to manage legume diseases caused by *R. solani*. However, chemical methods have severe health and environmental concerns [18]. Therefore, researchers are interested in developing non-chemical methods for the more efficient and sustainable management of legume diseases. Among the non-chemical methods, identifying and deploying resistant cultivars are now considered the most efficient and environmentally sound techniques to manage legume disease caused by *R. solani* [19]. However, when new management measures need to be created, including the cultivation of *Rhizoctonia*-resistant cultivars, the genetics of *Rhizoctonia* and its effect on isolate pathogenicity is vital to understanding.

*Rhizoctonia solani* is categorized as a species complex made up of distinct anastomosis groups (AGs), each of which contains communities with distinct morphogenetic variations [1,2,20]. So far, only 13 AGs have been identified based on hyphal anastomosis reactions, morphology, pathogenicity/virulence, and genetic similarity [14,17,21,22,23], while AG-B1 was thought to be 14th anastomosis groups; however, AG-B1 (a bridging isolate) was reclassified as AG-2-B1 [24].

To assess the genetic diversity and phylogeny of anastomosis groups (AGs) of *R. solani*, a variety of molecular markers were employed, including inter-simple sequence repeats (ISSR, simple sequence repeats (SSR), single nucleotide polymorphisms (SNPs), amplified fragment length polymorphism (AFLP), restriction fragment length polymorphisms (RFLP), randomly amplified polymorphic DNA (RAPD) markers, electrophoretic karyotype, DNA-DNA hybridization, and the sequence analysis of the rDNA ITS1-5.8S-ITS2 region. These diverse markers were utilized to evaluate and characterize the genetic variability and AGs of *R. solani*. Among these molecular markers, the sequence analysis of the rDNA ITS1-5.8S-ITS2 region stands out as a particularly potent tool for investigating genetic diversity and phylogenetic relationships within the anastomosis groups (AGs) and AG subgroups of *R. solani* [25,26,27,28,29,30,31,32,33,34]. The rationale is that all eukaryotic genomes contain these regions, and they are rapidly evolving and are surrounded by nucleotide sequences that are highly conserved. In addition, they also have incredibly high copy numbers, and the sequence alterations are much fewer [35]. Molecular analysis based on rDNA-ITS regions validates the traditional hyphal anastomosis methodology used to determine the grouping of AGs. The additional advantage is that they produce more detailed and precise patterns than the grouping of AGs through standard hyphal anastomosis reactions [34,36].

Despite the significance of legumes in the world and the significant economic threats of *Rhizoctonia solani* to legumes, there is no comprehensive worldwide investigation of genetic diversity and phylogeny of *R. solani* AGs associated with major grain legumes (soybean, chickpea, pea, cowpea, and beans) and forage legumes (alfalfa and clover). However, there are limitations in the present study. The availability of only a limited number of studies/sequences related to AGs associated with alfalfa, clover, cowpea, and peanut has resulted in uneven sample sizes. For instance, only AG-4 has been reported in alfalfa, causing seed rot and damping-off. Similarly, in clover, only AG-1, which leads to blight and sheath rot, has been identified, while in cowpea, it causes root and stem rot. In the case of peanuts, both AG-4 and AG-3 are responsible for root rots. Preliminary phylogenetic trees have been constructed to examine the distribution of AGs within each of these legume crops. However, in order to construct comprehensive phylogenetic trees encompassing all legume crops, the limited studies/sequences associated with alfalfa, clover, cowpea, and peanut have been excluded. Additionally, geographical factors may contribute to the grouping of AG sequences according to their host. However, our study reveals that sequences associated with legume crops tend to cluster based on AGs rather than geographical origin. Nevertheless, separating the effects of host and geographical origin is challenging. Hence, we have carefully curated our dataset to address these limitations. No previous efforts have been made to investigate the worldwide genetic diversity and distribution of *R. solani* associated with major grain legumes (soybean, chickpea, pea, cowpea, and beans) and forage legumes (alfalfa and clover). Given the genetic diversity and the varying disease-causing abilities of *R. solani* AGs on these legumes, the objectives of the current study were (1) to determine the number of studies/sequences associated with *R. solani* AGs associated with these legume crops; (2) to perform phylogenetic analysis and to identify the genetic diversity of rDNA ITS1-5.8S-ITS2 region sequences within each legume crop and across these legume crops; and (3) to identify the percentage of sequence identities within and between the clades of phylogenetic trees.

## 2. Results

### 2.1. Anastomosis Groups Sequences/Studies Associated with Legume Crops

The ITS sequences data pertaining to approximately 277 anastomosis groups (AGs) associated with major grain and forage legumes were extracted from the NCBI, as shown in Table 1 and Table S8. Among these sequences, AG-4, AG-2, and AG-1 sequences were the most prominently represented AGs, as indicated in Table S8 and Table 1. Soybean exhibited the highest number of reported AGs (more AG sequences associated with soybean are found in NCBI) compared to other legume crops (Table 1 and Table S1). AG-1 had the highest number of associated studies (sequences in NCBI), followed by AG-2, AG-4, AG-7, AG-11, AG-6, and AG-9. Within the subgroups, AG-1-IA was the most extensively investigated AG in soybean, followed by AG-2-2IIIB, AG-4-HGII, and AG-4-HGIII (Table 1). Thus far, only three AGs have been investigated in common bean, namely AG-2, AG-4, and AG-1. Among the subgroups, AG-2-2WB was the most commonly studied subgroup, followed by AG-4-HGI, AG-4-HGII, AG-1-1F, and AG-2-2IIB. AG-4-HGII, a subgroup of AG-4, was frequently reported in studies on chickpeas and peas. Limited research has been conducted on AGs associated with alfalfa, clover, peanuts, and cowpeas. For instance, AG-4-HGII and AG-1-1A were studied in alfalfa, while AG-1-IB and AG-11 were examined in clover. Similarly, AG-4-HG1, AG1-1A, AG4-1, and AG-5 were reported in peanuts. In the case of cowpeas, only AG-1-1A and two unidentified AGs have been studied (Table 1).

### 2.2. Phylogenetic Analysis of AGs within Each Legume Crop

**Figure 1:**
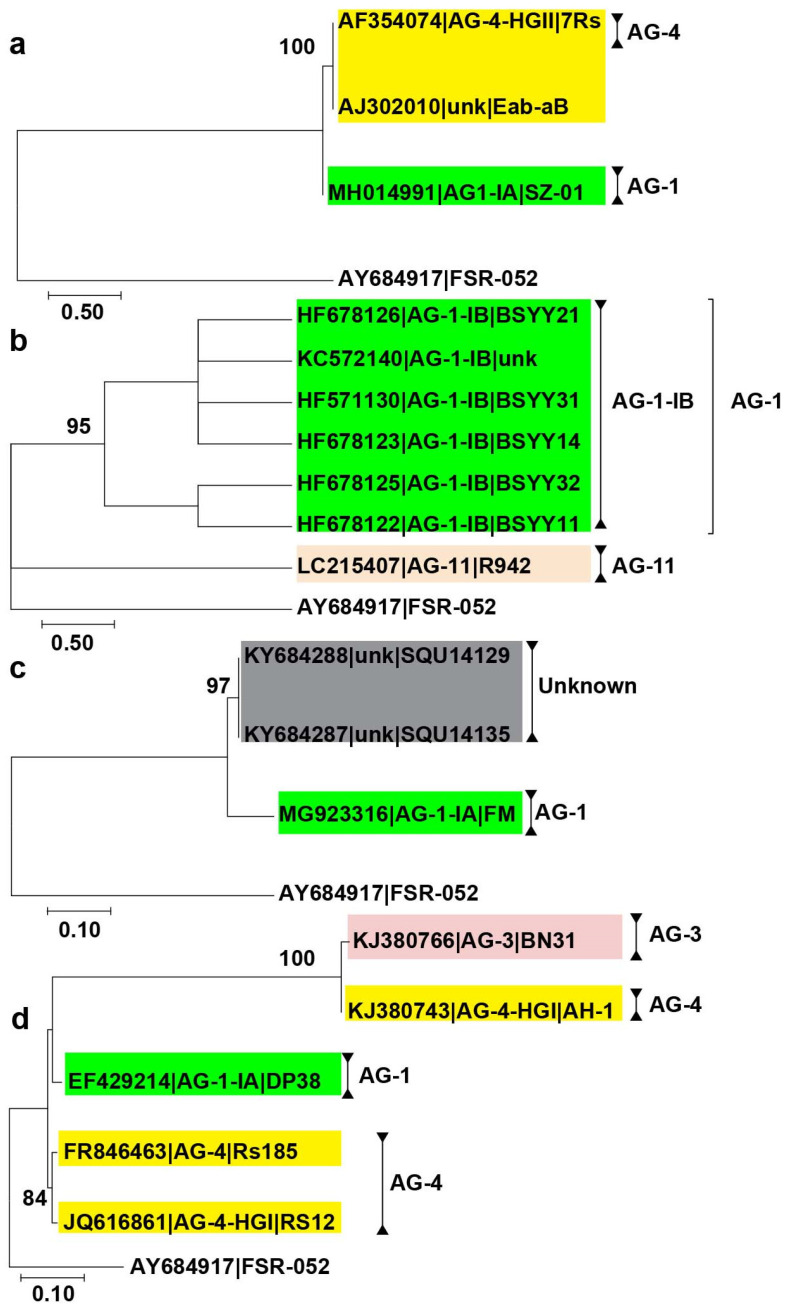
Phylogenetic trees of AG sequences associated with clover, alfalfa, cowpea, and peanut were constructed using the maximum likelihood (ML) method. Only bootstrap values ≥ 70% are shown. Accession number is followed by AGs from the GenBank and isolate name. Different colors show AGs and subgroups. (**a**,**b**). Few AG were reported from forage legumes such as alfalfa and clover, (**c**,**d**). AGs are associated with cowpea and peanut. Scale bar represents genetic distance for horizontal branch lengths. *Athelia rolfsii* (strain FSR-052) was used as an outgroup.

AGs associated with alfalfa are grouped into two discrete clades. One clade contains AG-4 isolates and the other clade includes AG-1 isolates (Figure 1a). Similarly, AGs associated with clover are split into two clades, one with AG-1-IB isolates and the other with AG-11-IB isolates (Figure 1b). AGs associated with cowpea, on the other hand, formed a single clade comprising two unknown AGs and AG-1-1A isolates (Figure 1c). Similarly, AGs associated with peanut are clustered into two clades, one clade corresponds to an isolate of AG-3, AG-1, and AG-4-HGI, and the other clade include AG-4 isolates (Figure 1d).

**Figure 2:**
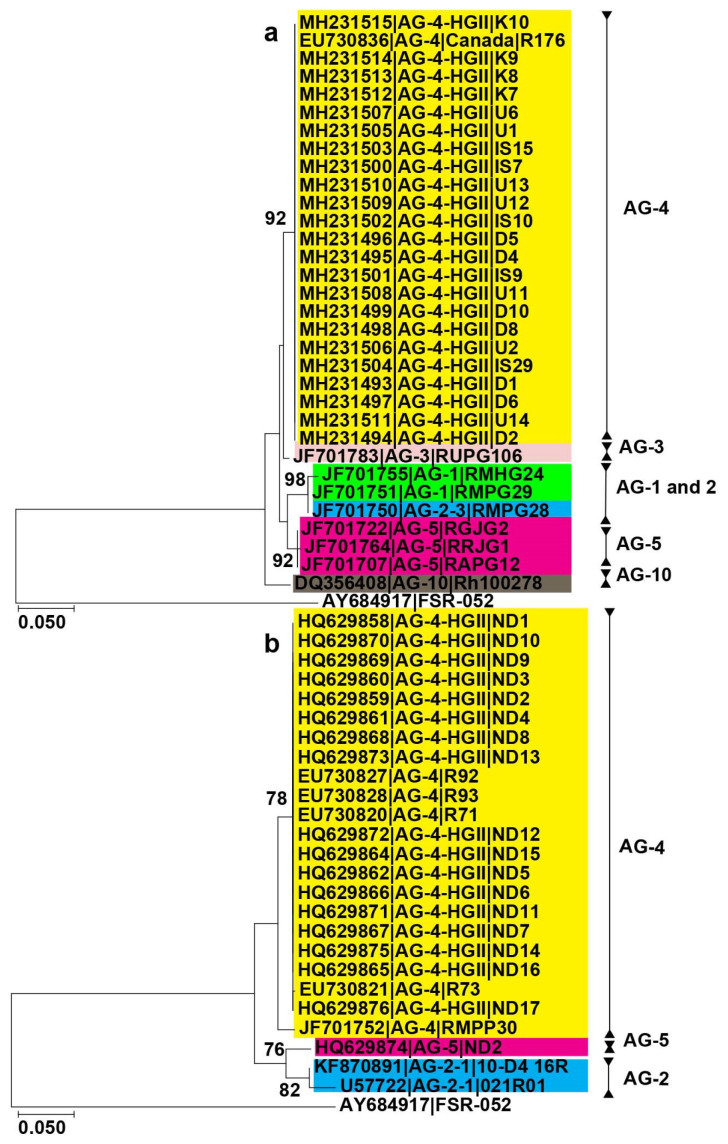
Phylogenetic trees of AG sequences associated with chickpea and pea were constructed using the maximum likelihood (ML) method. Only bootstrap values ≥ 70% are shown. Accession number is followed by AGs from the GenBank and isolate name. Different colors show AGs and subgroups. (**a**) AGs associated with chickpea; (**b**) AGs associated with pea. Scale bar represents genetic distance for horizontal branch lengths. *Athelia rolfsii* (strain FSR-052) was used as an outgroup.

**Figure 3:**
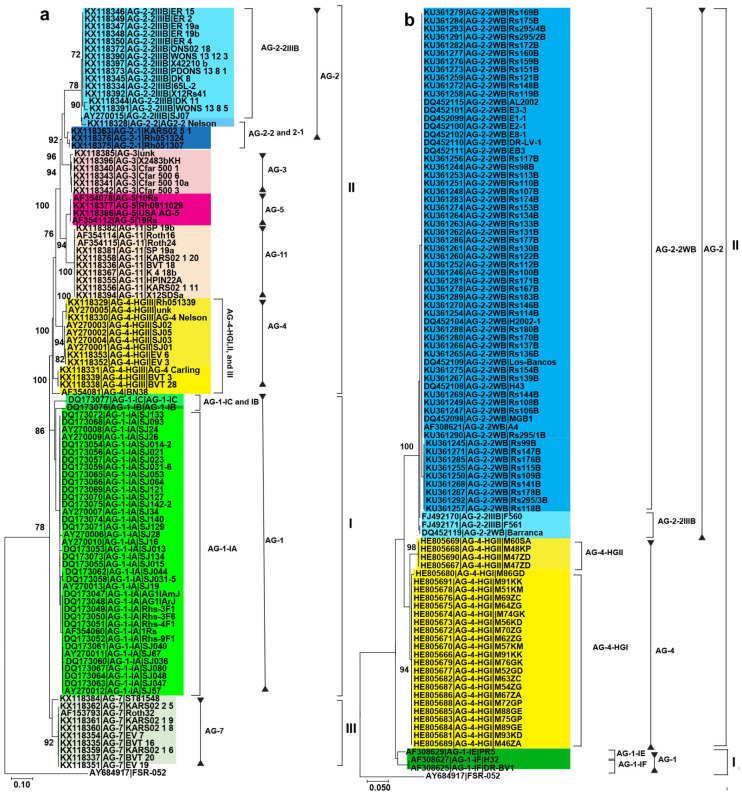
Phylogenetic trees of AG sequences associated with soybean and common bean were constructed using the maximum likelihood (ML) method. Only bootstrap values ≥ 70% are shown. Accession number is followed by AGs from the GenBank and isolate name. Different colors show AGs and subgroups and major clades associated with AGs, and these are represented by Roman numerals. (**a**) Phylogenetic tree of AGs associated with soybean; (**b**) Phylogenetic tree of AGs associated with the common bean. Scale bar represents genetic distance for horizontal branch lengths. *Athelia rolfsii* (strain FSR-052) was used as an outgroup.

Furthermore, AGs associated with chickpea exhibited clustering in two primary clades. The first clade consisted of a sole member, AG-10, while the second clade was subdivided into two subclades. The first subclade comprised isolates of AG-4 and AG-3, whereas the second subclade encompassed members of AG-1, AG-2, and AG-5 (Figure 2a). Similarly, AG associated with pea are clustered into two major clades, and one clade is further split into two subclades. One subclade corresponds to AG-4-HGII and AG-4 isolates. The other subclade contains AG-4 and AG-2-2 isolates. The other clade is further split into two subclades; one includes AG-2-1 isolates, while the other includes AG-5 isolates (Figure 2b). The AGs associated with soybean are grouped into three major clades (Figure 3a). One contains AG-1 isolates and is further split into distinct subclades corresponding to isolates of subgroups (AG-1, AG-1-1B, and IC), while the other contains subclades of AG-2, AG-3, and AG-4 isolates. Interestingly, AG-5 and AG-11 isolates form a sister subclade. The last clade corresponds to AG-7, isolates and is separate from the other two clades. Even AG subgroups within these subclades form distinct clusters. For example, AG-2-2 and AG-2-1 isolates did not cluster with AG-2-2IIIB isolates. Similarly, AG-4-HGIII isolates form a separate cluster than AG-4-HGI and AG-4-HGII isolates. Finally, AGs associated with the common bean form two major clades. One clade corresponds to AG-1 isolates (AG-IE and IF) and the other clade corresponds to AG-4 (HGI and HGII) and AG-2 (2WB and 2IIIB) isolates. Even subgroups of AGs cluster separately, for example, AG-2-2IIIB forms a sister subclade with AG-2-2WB. Similarly, AG-4-HGI forms a sister subclade with AG-4-HGII (Figure 3b). In conclusion, phylogeny analysis revealed that the isolates clustered consistently according to the AGs and subgroups within each legume crop.

### 2.3. Phylogenetic Analysis and Genetic Diversity of AGs across the Legumes

**Figure 4:**
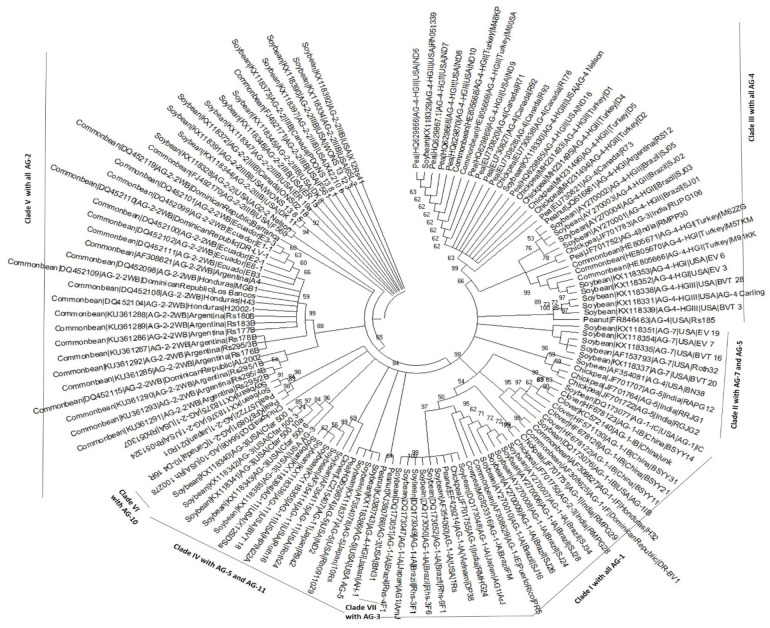
Phylogenetic trees of sequences associated with all legume crops were constructed using the maximum likelihood (ML) method. Only bootstrap values ≥ 70% have been shown. The common name of the legume crop is followed by reference accession numbers, AGs from the GenBank, and isolate name. Different colors show AGs and clades and/or subclades associated with AGs. The scale bar represents genetic distance for horizontal branch lengths. *Athelia rolfsii* (strain FSR-052) was used as an outgroup.

The preliminary analysis of the sequences using the NJ and ML method clustered AGs into three major distinct groups (Clades I, II, and IV) and three minor groups (Clades III, V, and VI) (Figure S1). Clade I encompasses AG-1 isolates, Clade II has only AG-2 isolates, and Clade IV has only AG-4 isolates. Clade III is a distinct group containing only AG-3 isolates, and Clade V consists of AG-5, AG-11, and AG-7 isolates. AG-3, AG-4, and AG-1 isolates associated with alfalfa and peanut form Clade VI (Figure S1). These AGs isolates should fall into their respective AG groups. For example, AG-3 and AG-4 isolates should fall in Clade III and Clade IV. Few sequences/studies have been conducted on *R. solani* AGs associated with alfalfa, clover, cowpea, and peanut. AGs associated with these legumes fall in Clade VI (Table S1). Hence, the AG of Clade VI will not be incorporated in the subsequent studies because the limited studies/sequences indicate low sample size, and these could behave as outliers in the phylogenetic trees. Additionally, this preliminary phylogenetic tree was huge. Accordingly, some of the AGs subgroups within the subclades, belonging to the same host legume crop, were removed to make it more transparent. The sequences were again analyzed, as shown in Figure 4; consequently, isolates split into seven clades: Clade I, Clade II, Clade III, Clade IV, Clade V, Clade VI, and Clade VII. Clade I encompasses AG-1 isolates, Clade II encompasses AG-5 and AG-7 isolates, Clade III encompasses AG-4 isolates, Clade IV encompasses AG-11 and AG-5 isolates, Clade V encompasses AG-2 isolates, Clade VI encompasses AG-10 isolates, and Clade VII encompasses AG-3 isolates (Figure 4). Within Clade I, isolates of chickpea, soybean, peanut, and common bean (subgroups IA and IE) form a distinct subclade from the isolates of clover and soybeans (subgroups IF, IB, and IC). Also, subgroups IF and IB form two subclades, whereas subgroup IC forms a separate clade from subgroups IA, IE, IFs and IB. Within Clade II, three chickpea (AG-5) isolates and one soybean isolate (AG-5) form a sister clade with AG-7 isolates. Within Clade IV, a few isolates of soybean and pea (AG-5) also form a sister clade with isolates of clover and soybeans (AG-11).

Bootstrap analysis determined that subgroups HG-I, HG-II, and HG-III within AG-4 were located on statistically significant branches within Clade III. Furthermore, the isolates from soybean (AG-4-HGI) and common bean (AG-4-HGI) cluster together. Similarly, isolates from pea (AG-4-HGII) and isolates from chickpea (AG-G-HGII) cluster together. Likewise, within Clade V, isolates from soybean and pea (AG-2-2-1) formed a group separate from the rest of the subgroups (AG-2-2WB, AG-2-IIIB, and AG-2-2). Clades VI and VII include AG-10 and AG-3 isolates, respectively (Figure 4). A few AGs or subgroups isolates were in the wrong position in the phylogenetic tree (Figure 4). For example, one isolate (BN38) of AG-4 was associated with soybean clusters with AG-5 isolates rather than isolates of AG-4. Also, an AG-2-1 (RMPG28) isolate clustered with AG-1 isolates rather than AG-2 isolates.

### 2.4. Percent Sequence Identities within and between Clades

The range of percent rDNA-ITS sequence identity within and between the clades (I to VII) of the phylogenetic tree (Figure 4) is summarized in Table 2. The percent sequence identity within Clade I (AG-1 isolates) ranged from 78.9% to 100%. AG-1, AG-1-1A, and AG-1-IE isolates had higher sequence identities (91.5–100%) than AG-1-IF, AG-1-IB, and AG-1-IC (80.4–90.5%). This suggests a tighter genetic relationship between the AG-1, AG-1-1A, and AG-1-IE isolates. Furthermore, Clade II includes AG-5 and AG-7 isolates; the sequence identity of the isolates within this clade is greater than 90.5%. Several AG-5 isolates also form a clade with AG-11 isolates, and the sequence identity of the isolates within this clade is greater than 71%. Likewise, within Clade III, AG-4-HG1, AG-4-HGII, and AG-4-HGII isolates had a 91–100% sequence identities range. Similarly, within Clade V, the sequence identities between AG-2-1 and AG-2-2WB, AG-2-1 and AG-2-2IIIB, and AG-2-1 and AG-2-2 were 70–80%, 74–82%, and 74.8–82%, respectively. Whereas sequence identities between AG-2-2WB and AG-2-2IIIB, AG-2-2 and AG-2-2WB, and AG-2-2 and AG-2-2IIIB were 95–97%, 96.1–96.3%, and 96.3-97%, respectively. This indicates closer genetic relatedness between AG-2-2, AG-2-IIIB, and AG-2-2WB isolates than isolates of AG-2-1, and it is shown in the phylogenetic tree below that isolates of AG-2-1 form a separate subclade than the isolates of AG-2-2, AG-2-WB, and AG-2-IIIB. Sequence identities ranged from 55.8 to 100% among the clades (Table 2). Clade 1 (AG-1 isolates), Clade III (AG-4 isolates), and Clade V (AG-2 isolates) were the major clades in the phylogenetic tree (Figure 4). The sequence identities between Clade I and III (80.7–86.5%) were relatively higher than those between Clades I and V (67.5–81.6%). Similarly, the sequence identities between Clades III and V were 72.7–84.7% (Table 2). This indicates closer genetic relatedness between Clade I (AG-1) and Clade III (AG-4) than the isolates of Clade V (AG-2).

### 2.5. Principal Coordinate Analysis of Anastomosis Groups across Legumes

**Figure 5:**
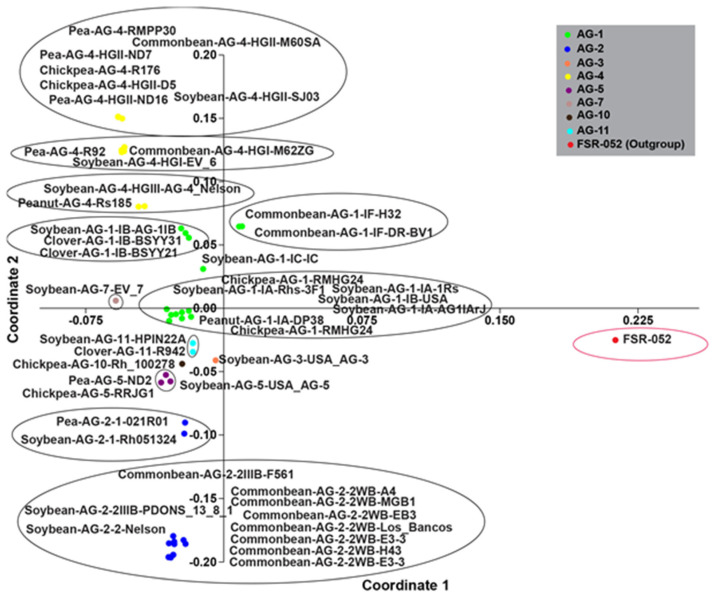
Principal coordinate analysis (PCoA) scatter plot based on the percent sequence identity matrix. Different colors of plots represent different AG groups associated with legume: AG-1 (green circle), AG-2 (blue circle), AG-3 (bisque circle), AG-4 (yellow circle), AG-5 (purple circle), AG-7 (antique white circle), AG-11 (aqua circle), AG-10 (brown circle), and FSR-052 (outgroup—red circle).

PCoA showed that AGs clustered without being related to their host legume crops (Figure 5). Moreover, AGs clustered into subgroups, and even the subgroups of an AG form separate clusters. For example, subgroups HG-I, HG-II, and HG-III of AG-4 form separate clusters. Similarly, the isolates of common bean and soybeans (AG-2-2WB, AG-2-2, and AG-2-2IIIB) form a distinct cluster separate from the isolates of soybean and peas (AG-2-1). This indicates that sequences of AG-2-2WB, AG-2-2, and AG-2-2IIIB are more closely related to each other than to AG-2-1. Moreover, isolates from chickpea, common bean, soybean, and peanut (AG-1-1A and AG-1-IE) form a distinct cluster separate from the clusters consisting of isolates AG-1-IF, AG-1-IB, and AG-1-IC. Hence, the sequences of AG-1-IA and IE were more closely related to each other than the sequences of AG-1-IB, AG-1-IF, and AG-1-IC (Figure 5).

## 3. Discussion

*Rhizoctonia solani* is a severe soil-borne pathogen that affects various crops worldwide and has a significant economic impact in all locations where legume crops are grown [50]. Most of the *R. solani* AG sequences collected from NCBI associated with legume crops in this study belonged to AG-1, AG-2, and AG-4 (Table S8).

The findings of our study reveal a notable disparity in the availability of AG sequences associated with different legume crops in the NCBI database. Specifically, a greater abundance of AG sequences was found for soybean, chickpea, common bean, and pea, while comparatively fewer AG sequences were associated with alfalfa, peanut, clover, and cowpea. This discrepancy in the representation of AG sequences across legume crops suggests variations in the extent of research focus and sequence data availability for different crops within the legume family. Soybean, chickpea, common bean, and pea, being economically important crops, have likely received more attention in terms of AG characterization and sequence data deposition in public databases such as NCBI. These crops may have been the subject of more extensive studies exploring the diversity and genetic relationships within their AG populations. On the other hand, the relatively limited number of AG sequences associated with alfalfa, peanut, clover, and cowpea may be indicative of a lesser emphasis on AG studies for these particular crops or a scarcity of available sequence data in the NCBI database. Additionally, few AGs associated with alfalfa, peanut, clover, and cowpea indicate that these legumes are susceptible to specific AGs, whereas more AG studies on soybean, pea, common bean, and chickpea indicate that these legumes are susceptible to a wide arrays of AGs [51,52]. The kinetics of the host–pathogen interaction, genetic flexibility, level of adaptation, and spread are additional factors that have an impact on the diversity, frequency, and spread of AGs [15]. Other factors, such as crop rotation and climatic factors, may also prefer the prevalence of certain AGs over others [53,54]. Consistent with prior studies, our findings support that AG-1, AG-2, and AG-4 have received greater research attention compared to other anastomosis groups (AGs) in legumes. This is evidenced by the higher representation of sequences associated with AG-1, AG-2, and AG-4 in the NCBI database. The abundance of sequences for these AGs suggests that they have been the focus of numerous investigations, resulting in a larger pool of available data [13,15,17,20,24,28,30,40,41]. AG-2 has a worldwide distribution and causes damping-off in many plants, brown patches of turfgrass and cereals, and root rot in several grain legume crops [1,42]. AG-4 is also distributed worldwide and has a broad host range [23]. AG-1 is reported to cause blights (aerial and web), seed, root, and stem rots of legume crops [15,25,43,44].

Moreover, our study reveals that closely related anastomosis groups (AGs) associated with legumes tend to cluster together, irrespective of their geographical origin (Figure S1). This finding supports the notion that AGs are more influenced by genetic relatedness than by the specific geographic location from which they have been identified. The observed clustering patterns suggest that AGs associated with the same legume species share a closer evolutionary relationship, regardless of whether they were isolated from different geographic regions [55,56,57,58].

Also, there were variations in the number of sequences on the subgroups of AGs. For example, subgroups of AGs, such as more sequences of AG1-1A associated with soybean are found in NCBI followed by AG-2-IIIB and AG-4, AG-2-2WB. AG-2IIIB and AG-4 cause damping-off, root, and hypocotyl rot in soybeans, while AG-1-IA and AG-2-IIIB cause foliar blights [12]. One of the most severe diseases of soybean, leaf blight, is caused in particular by AG-1-IA, which is significant on a global scale [30,59]. Similarly, AG-2-2WB-caused web blight is one of the most devastating diseases of the common bean globally, and AG-2-2WB has frequently been retrieved from the common bean [15]. Likewise, AG-4 subgroups cause severe root, hypocotyl, crown rot, and the damping-off of chickpea, pea and peanut and have been frequently recovered from these legume crops [59,60]. The dominance of specific AGs in a legume crop is likely because that AG is more pathogenic to the legume crops grown in that geographical region and, therefore, frequently recovered from that legume crop [61].

*Rhizoctonia solani* have been classified into AGs and subgroups using various molecular methods [34,36,46,47]. The sequence analysis of the rDNA-ITS region has drawn the most interest for the phylogenetic analyses of *R. solani* AGs and their subgroups [21,48,49,50,51] and are frequently employed to quickly identify and categorize *R. solani* AGs. These rDNA-ITS regions have been used to study the genetic diversity in many fungi, including *R. solani,* and the development of species-specific probes [55,62].

In this study, the phylogenetic analyses of *R. solani* rDNA-ITS sequences associated with legume crops revealed that AGs and their subgroups form distinct clades or subclades. For example, in the case of soybean, AG-1, AG-2, AG-3, AG-4, AG-10, and AG-7 form distinct clades or subclades. Even within each subclade or clade, subgroups are separate from each other. For example, AG-1C and IB isolates form a sister subclade with AG-1A and AG-IE isolates within the AG-1 clade. Moreover, the AG-2-2 isolates constitute a distinct group from the AG-2-1 isolates within the AG-2 clade. The AG-2 isolates associated with other crops are thought to be polyphyletic, with subgroups reliably creating distinct clades or subclades [63]. Similarly, in the case of common beans, two major clades associated with AG-1, AG-2, and AG-4 were noted. Even within the AG-2, AG-2-2IIIB isolates form a sister clade with AG-2-2WB isolates. Furthermore, within the AG-4 clade, AG-4-HGIII isolates form a sister clade with AG-4-HGII and AG-4-HGI isolates. Previous studies have shown that subgroups HGI and HGII are more related to each other than to subgroup HGIII [28,57]. Our study revealed that AGs form distinct clades or subclades within each legume crop, but this can be true for soybean, chickpea, common bean, and pea because the rDNA-ITS sequence data on Ags associated with these legume crops are sufficiently available in GenBank and publications. However, only a few available sequences of AGs associated with alfalfa, cowpea, peanut, and clover are available in GenBank. Authors of the reference [64] stated that a sequence of only one or few isolates is available for Ags, which may render the accuracy and location of these Ags in the phylogenetic tree [36]. In addition, the use of GenBank rDNA-ITS sequences for the analysis of only one or a few isolates can occasionally be misleading. In conclusion, each AG formed single and independent groups or clades, and these results also agree with previous studies [17].

Moreover, in this study, the phylogenetic analysis across the legumes revealed that most of the Ags and/or their subgroups rarely separate, corresponding to their legume host crop (Figure 4). For example, AG-4-HGII isolates of pea, chickpea, common bean, peanut, and soybean cluster together and form distinct subclades. Likewise, AG1-IA isolates of chickpea, soybean, common bean, and chickpea cluster together and form distinct subclades. Similarly, AG-2-1 isolates of soybean and pea formed a group separated from AG-2-2WB and AG-2-IIIB isolates of common bean and soybean. The distinct clades on the phylogenetic trees in this study agree with previous studies and prove that AGs/subgroups of *R. solani* are positioned on the evolutionary tree independently from each other [40]. Nonetheless, a degree of host specificity is also observed among AGs in the phylogenetic tree. The authors of [59,60] observed a certain degree of host specificity among AGs [65]. Some of the sequences of AGs associated with legumes were also located in the wrong position in the phylogenetic tree (Figure 4). For example, one isolate (RMPG28) of AG-2-1 associated with chickpea was located within Clade I (clade of AG-1 isolates). Likewise, one isolate (RUPG106) of AG-3 associated with chickpea was located within Clade II (AG-4 isolates clade). In addition, one isolate (BN38) of AG-4 associated with soybean formed a group with AG-5 isolates associated with chickpea rather than isolates of Clade II (AG-4 isolates clade). Previous studies indicate that the inaccurate sequences of unknown AGs, according to their locations in the phylogenetic trees, can be deposited in the GenBank [62].

Additionally, the based on genetic similarities pairwise distance matrix showed that the isolates within the clades and subclades are more similar than the isolates of other clades. In addition, PCoA revealed that different subgroups of an AG form a separate group from each other. For example, the AG-1-IF, AG-I-IB, and AG-I-IC isolates form separate clusters to those of AG-IE and AG-IA isolates. It indicates that isolates of IE and IA were more identical than IF, IB, and IC subgroups of AG-1. According to earlier studies, isolates from the same subgroup had higher genetic similarity in the ITS regions than isolates from various subgroups within the same AG and isolates from different AGs [34]. Hence, all AGs and their subgroups were positioned independently, and the clustering of AGs did not correspond to the host legume crop. The authors of [66] found that *R. solani* isolates were independent or did not correspond to their host crop.

## 4. Materials and Methods

### 4.1. Data Collection

The major grain legumes, such as pea (*Pisum sativum*), common bean (*Phaseolus vulgaris*), chickpea (*Cicer arietinum*), peanut (*Arachis hypogaea*), cowpea (*Vigna unguiculata*), and soybean (*Glycine max*), and forage legumes (*Medicago sativa*), such as clover (*Trifolium* spp.), were targeted. The following parameters were used to obtain the rDNA-ITS sequences of *R. solani* isolates from these legume crops of the National Center for Biotechnology Information (NCBI) GenBank (https://www.ncbi.nlm.nih.gov/genbank; accessed on 10 November 2022): (1) the data of these sequences were published in peer-reviewed journals from 2001 to 2022 and can be downloaded from NCBI; (2) isolates had available information on geographical origin, legume host crop, isolates, AGs, and pathogenicity; and (3) unknown but unique AGs associated with the legume crops were also selected. The rDNA ITS1-5.8S-ITS2 region in this study is a specific region within the ribosomal DNA (rDNA) gene cluster. It includes the internal transcribed spacer 1 (ITS1), the 5.8S ribosomal RNA gene, and the internal transcribed spacer 2 (ITS2). The rDNA gene cluster is composed of several repeating units, each containing the 18S ribosomal RNA (rRNA) gene, the ITS1 region, the 5.8S rRNA gene, the ITS2 region, and the 28S rRNA gene. The ITS1 and ITS2 regions lie between the 18S and 5.8S rRNA genes and between the 5.8S and 28S rRNA genes, respectively [34,35,36]. The length of this region was around 500 to 700 base pairs (bp) in length [34,35,36].

### 4.2. Number of Studies That Report Anastomosis Groups

A comprehensive literature search using the internet facility of Karakoram International University (KIU) Gilgit, Pakistan, was conducted using various academic databases, including Google Scholar (http://scholar.google.com; accessed on 11 December 2022), Web of Science (http://apps.webofknowledge.com; accessed on 21 December 2022), PubMed (https://pubmed.ncbi.nlm.nih.gov/; accessed on 25 December 2022), and Scopus (https://www.elsevier.com/en-gb/solutions/scopus; accessed on 2 January 2023). The search utilized the primary keywords “legumes-*R. solani*” and “legumes-anastomosis groups (AGs)” both independently and in combination. The search was limited to articles published between January 2001 and November 2022. To be eligible for inclusion in this study, the selected literature had to satisfy the following criteria: (i) only peer-reviewed journal articles were considered; (ii) articles explicitly mentioning AG accession numbers and sequencing data that were publicly available in NCBI GenBank were included; (iii) articles discussing the geographical origin, isolates, and AGs causing symptoms in legumes were included; and (iv) articles encompassing pathogen isolation from various sources, such as rhizosphere soil, topsoil, roots, and shoots of symptomatic legume crops, were included. Approximately seventy-six relevant articles were identified and retrieved from the aforementioned databases. These articles were imported into the EndNote X9 software to facilitate the compilation of information regarding AGs, isolates, geographical origins, and the sources of isolation. Metadata on isolation sources, isolates, geographical origin, and the pathogenicity of AGs were compiled for all the sequences of AGs with references (Tables S1–S6). The primary isolation sources included blights (foliar/web), rots (seeds, stems, roots, crowns, hypocotyls, and collars rots), and the damping-off of the legume crops. The number of studies that report AGs associated with legume crops was also recorded.

### 4.3. Sequences Alignments

The sequences were assembled using BioEdit v. 7.2.5 [67,68]. The sequences were then aligned using MAFFT v. 7.471 (https://mafft.cbrc.jp/alignment/server/index.html, accessed on 15 May 2023), which enables the quick identification of homologous regions using fast Fourier transform (FFT) through the repeated improvement of original alignments [68]. The aligned sequences were uploaded into BioEdit v. 7.2.5 [67] for concatenation and trimming ambiguously aligned regions.

### 4.4. Phylogenetic Analysis

Data regarding AGs, isolate names, GenBank accession no, and geographical origins with references are shown in Tables S1–S6. The most effective models of sequence evolution, as shown in Table S7, were chosen before phylogenetic analysis using jModelTest v. 2.1.6 [69] and the Molecular Evolutionary Genetics Analysis program (MEGA X) [69] package program following the criteria of the Bayesian test (BAY) and Akaike information criterion (AIC) [41]. The Tamura-Nei [70] and Tamura 3-parameter [68] models were utilized via MEGA-X and jModelTest. Phylogenetic trees obtained from the aligned sequences using maximum likelihood (ML) [71] and neighbor-joining (NJ) [72] were constructed using MEGA X. All trees were rooted with *Athelia rolfsii* FSR-052 (GenBank Accession No. 132 AY684917). G (gamma distributed) rates were selected for both ML and NJ, and uniform rates were also selected for NJ. Nearest-neighbor interchange (NNI) was chosen as the heuristic procedure, and the partial elimination was configured as a gap/missing data treatment with a 95% site coverage threshold. ML analysis was also performed with RAxML v. 8.2.12 [73]. Each phylogenetic tree was put through a bootstrapping test, which involved drawing 1000 random samples from different aligned sequences. Only nodes with 70% or greater bootstrap values were displayed on the phylogenetic trees. The web program iTOL (http://iTOL.embl.de/, accessed on 15 May 2023) [74] and FigTree v. 1.4.4 (http://tree.bio.ed.ac.uk/software/figtree/, accessed on 15 May 2023) were used to display the phylogenetic trees. The phylogenetic trees were downloaded and edited in Adobe Illustrator CS5.

### 4.5. Principal Coordinate Analysis (PCoA)

Sequence identities among the various isolates within and between AGs and subgroups were determined using BioEdit version 7.2.5. [75]. The investigation of sequence identities within and between AGs and subgroups, as well as among them, was carried out using principal coordinate analysis (PCoA) with the Gower index, conducted using PAST version 4.03 (a statistical software package designed for data analysis and education in paleontology) [76].

## 5. Conclusions, Drawbacks, and Future Perspectives

In conclusion, this research is the first to provide comprehensive data on the genetic diversity and phylogeny of *R. solani* AG associated with legume crops. It is important to note that the availability of AG sequences in public databases can influence the accuracy and comprehensiveness of phylogenetic analyses and evolutionary studies involving *R. solani* and its interactions with different legume crops. Future research endeavors should aim to expand the dataset of AG sequences for underrepresented legume crops, such as alfalfa, clover, cowpea, and peanuts, enabling a more inclusive and comprehensive analysis of the genetic relationships and evolution of *R. solani* across the legume family. Our study highlights the imbalance in AG sequence representation across different legume crops in the NCBI database. This emphasizes the need for further investigations and data acquisition to enhance our understanding of AG diversity and its implications for the management and control of *R. solani* in a broader range of legume crops. The phylogenetic analysis of AGs revealed that closely related anastomosis groups (AGs) associated with legumes tend to cluster together, irrespective of their geographical origin and host legume crop. This finding supports the notion that AGs are more influenced by genetic relatedness than by the specific geographic location and/host from which they have been identified. The observed clustering patterns suggest that AGs associated with the same legume species share a closer evolutionary relationship, regardless of whether they were isolated from different geographic regions. A few isolates of *R. solani* associated with legumes were located in the wrong position in the phylogenetic tree, indicating inaccuracies in the designation of AGs or subgroups deposited in GenBank. Although the analysis of the rDNA-ITS sequences has been proven to be a reliable approach for the phylogenetic studies of AGs and subgroups in *R. solani* associated with legume crops, some limitations exist. The sequence data deposited in the repositories and databases are not being validated or verified by other researchers; therefore, incorrectly named AGs is almost expected. Sometimes, incomplete information regarding AGs, such as the proper isolate name or geographical origin, is not mentioned. Therefore, additional sequences from other DNA regions need to be incorporated to determine the genetic diversity and the phylogeny of AGs of *R. solani*. These additional sequences will assist in confirming new AGs groups or subgroups associated with legume crops. Moreover, it will also make it possible to understand the diseases of legumes caused by *R. solani* and help develop novel management methods to suppress the diseases of legumes.

## Figures and Tables

**Table 1:** Number of sequences/studies in which anastomosis groups (AGs) or subgroups of *Rhizoctonia solani* associated with leguminous plants are found.

Legume Crop	Group	^b^ Subgroup	Number of Sequences/Studies
Alfalfa	^a^ AG1	IA	1
	AG4	HGII	2
Clover	AG1	IB	6
	AG11	NA	1
Cowpea	AG1	IA	1
	Unc	2	2
Peanut	AG1	IA	1
	AG3	3	1
	AG4	4	1
	AG4	HGI	2
Common beans		AG1-IE	1
		AG1-IF	2
	AG2	AG2-2 WB	63
		2IIIB	2
	AG4	HGI	22
		HGII	4
Pea	AG2	AG2-1	2
		AG2-2	1
	AG4	AG4	5
		HGII	17
	AG-5	AG-5	1
Soybean	AG-1	1	1
		IA	38
		IB	1
		IC	1
	AG-2	1	3
		2	1
		2IIIB	18
		3	2
	AG-3	3	6
	AG-4	4	1
		HGI	2
		HGII	4
		HGIII	3
	AG-5	^c^ NA	4
	AG-6	^c^ NA	1
	AG-7	^c^ NA	10
	AG-9	^c^ NA	1
	AG-11	^c^ NA	10
Chickpea	AG-1	1	2
		3	2
	AG-3	3	2
	AG-4	4	1
		HGII	23
	AG-5	^c^ NA	2
	AG-10	^c^ NA	1
[37,38,39,40,41,42,43,44,45,46,47,48,49]			

^a^ AGs = anastomosis groups, ^b^ Sub. = AGs subgroup, ^c^ NA = not applicable/available, Unc = unclassified.

**Table 2:** Percent sequence identity of AGs within and between the clades of *Rhizoctonia solani* across the legume crops.

	CLADES																				
	I (AG-1)						II		III (AG-4)				IV		V (AG-2)				VI	VII	Outgroup
	IA	IB	IE	IF	IC	1	AG-5	AG-7	HG-1	HG-II	HG-III	4	AG-5	AG-11	AG-2-1	AG-2- 2WB	AG-2- 2IIIB	AG-2-2	AG-10	AG-3	
**IA**	95.6–100																				
**IB**	82.2–84.1	99.5–99.7																			
**IE**	96.1–100	84.1–84.6	99–100																		
**IF**	78.9–86.6	90.5–91.2	81.4–81.6	99–99.7																	
**IC**	86.4–87.6	82.9–83.1	84.2–86	80.4–80.6	99–100																
**1**	91.5–99.5	83.7–84.1	94.1–97.0	83.4–84.6	82.7–87.6	99–100															
**AG-5**	77.6–88.8						97.7–98	97.1–98													
**AG-7**	80.1–89.7						90.5–91.9	97–100													
**HG-1**	81.1–85.4						85.2–85.6	85.7–85.9	99–100												
**HG-II**	81.0–86.5						85.4–86.1	85.4–86.1	96–99	96–100											
**HG-III**	80.7–86.3						86.1–86.7	86–86.4	91–96	91–99	91–100										
**4**	82.2–86.5						85.4–86.4	85.4–86.1	96.2	96–100	92–96.2	97.9–100									
**AG-5**	72.6–82.6						81.7–86.4	82–86.8	78.4–85.2				94.7–99.5								
**AG-11**	75.2–80.8						83–84.1	82.5–84.6	80.3–84				89.1–93.8	91.3–100							
**AG-2-1**	67.5–77.8						71–80.6	76.4–84.9	72.7–84.7				80.7–90.7	78.4–88.2	90–99.2						
**AG-2-** **2WB**	74.5–81.6						83.3–83.6	82.6–84	79.1–79.9				79.8–84.5	81.5–82.7	70–80	98.6–99.7					
**AG-2-** **2IIIB**	77.2–81.3						83.3–84.2	82.3–82.7	79.5–80				79–84.4	81.5–83.9	74–82.1	95.6–97	97.9–99.5				
**AG-2-2**	74.5–80.7						83.3–84.6	82.1–82.2	78.5–79.6				78.5–83.2	80.9–82.3	74.8–82	96.1–96.3	96.3–97	98.2–99.0			
**AG-10**	73.3–81.7						83.2–84	83–84.5	81.4–84.3				85.5–89.7	85.7–87.5	81.4–82.4				99.9–100		
**AG-3**	67–76.2						74.8–80.1	75.4–85.9	70.9–82.1				80.2–89.9	81.4–89	55.8–76.9				90.2–91.5	96.8–99.2	
**Outgroup**	54–58						58	59	54–58				50–54	55.1–55.3	57–58				54	55	100

## Data Availability

The data is contained within the manuscript and supplementary materials.

## Supplementary Materials

The following supporting information can be downloaded at: https://www.mdpi.com/article/10.3390/plants12132515/s1, Figure S1: Phylogenetic trees of AG sequences associated with all legume crops using the Neighbor-Joining (NJ) method; Table S1: Data regarding anastomosis groups of R. solani associated with alfalfa and clover; Table S2. Data regarding anastomosis groups of R. solani associated with cowpea and peanut; Table S3. Data regarding anastomosis groups of R. solani associated with chickpeas; Table S4. Data regarding anastomosis groups of R. solani associated with common beans; Table S5. Data regarding anastomosis groups of R. solani associated with pea; Table S6. Data regarding anastomosis groups of R. solani associated with soybean; Table S7. Best substitution models used in this study; Table S8. Number of anastomosis groups (AGs) sequences of R. solani associated with legumes crop.
