# Supplementary material for: Assessment of Genetic Variability and Evolutionary Relationships of Rhizoctonia solani Inherent in Legume Crops
Source: Plants (Basel). 2023 Jun 30;12(13):2515. doi: 10.3390/plants12132515 (PMC10347096; doi:10.3390/plants12132515)
Supplement: Supplementary file 1 [file plants-12-02515-s001.zip › plants-2401632-supplementary.pdf]

Supplementary Materials

Table S1. Data regarding anastomosis groups of *R. solani* associated with alfalfa and clover.

S. No	Common name	Anastomosis Group	Accession numbers	Isolates	Patho-genicity	Geographic origin	References
1	Alfalfa	AG4-HG-II	AF354074	7Rs	Seed rot and damp-ing-off	USA	[20]
2	Alfalfa	AG4-HG-II	AJ302010	Eab-aB	"	Spain	"
3	Alfalfa	AG1-IA	MH014991	SZ-01	Root rot	China	[70]
4	Clover	AG11	LC215407	R942	Blight and sheath rot	Japan	[71]
5	Clover	AG1-IB	HF678126	BSYY21	Summer blight	China	[17]
6	Clover	AG1-IB	HF678125	BSYY32	"	China	"
7	Clover	AG1-IB	HF678123	BSYY14	"	China	"
8	Clover	AG1-IB	HF678122	BSYY11	"	China	"
9	Clover	AG1-IB	HF571130	BSYY31	"	China	"
10	Clover	AG1-IB	KC572140	AG-1-IB	"	China	"

Table S2. Data regarding anastomosis groups of *R. solani* associated with cowpea and peanut.

S. No	Common name	Anastomosis Group	Accession numbers	Isolates	Pathogenicity	Geographic origin	References
1	Cow pea	unk	KY684288	SQU141 29	Root and stem rot	Oman	[13]
2	Cow pea	unk	KY684287	SQU141 35	Root and stem rot	Oman	[13]
3	Cow pea	AG1-IA	MG923316	FM	Leaf blight	Brazil	[72]
4	Peanut	AG4	FR846463	Rs185	Root rot	USA	[73]
5	Peanut	AG1-IA	EF429214	DP38		Vietnam	Un-published
6	Peanut	AG3	KJ380766	BN31		USA	Un-published
7	Peanut	AG4-HGI	KJ380743	AH-1		Japan	Un-published
8	Peanut	AG4-HGI	JQ616861	RS12		Argentina	Un-published

Table S3. Data regarding anastomosis groups of *R. solani* associated with chickpeas.

S. No	Anastomosis Group	Accession numbers	Isolates	Pathogenicity	Geographical origin	References
1	AG4-HGII	MH231515	K10	Root and collar rot	Turkey	[24]
2	AG4-HGII	MH231514	K9	Root rot	Turkey	"
3	AG4-HGII	MH231513	K8	"	Turkey	"
4	AG4-HGII	MH231512	K7	"	Turkey	"
5	AG4-HGII	MH231511	U14	"	Turkey	""
6	AG4-HGII	MH231510	U13	"	Turkey	
7	AG4-HGII	MH231509	U12		Turkey	"
8	AG4-HGII	MH231508	U11	"	Turkey	"
9	AG4-HGII	MH231507	U6	"	Turkey	"
10	AG4-HGII	MH231506	U2	"	Turkey	"
11	AG4-HGII	MH231505	U1	"	Turkey	"
12	AG4-HGII	MH231504	IS29	"	Turkey	"
13	AG4-HGII	MH231503	IS15	"	Turkey	"
14	AG4-HGII	MH231502	IS10	"	Turkey	"
15	AG4-HGII	MH231501	IS9	"	Turkey	"
16	AG4-HGII	MH231500	IS7	"	Turkey	"
17	AG4-HGII	MH231499	D10	"	Turkey	"
18	AG4-HGII	MH231498	D8	"	Turkey	"
19	AG4-HGII	MH231497	D6	"	Turkey	"
20	AG4-HGII	MH231496	D5	"	Turkey	"
21	AG4-HGII	MH231495	D4	"	Turkey	"
22	AG4-HGII	MH231494	D2	"	Turkey	"
23	AG4-HGII	MH231493	D1	"	Turkey	"
24	AG3	JF701783	RUPG106	Web/foliar blight/wet root rot	India	[14]
25	AG5	JF701764	RRJG1	"	India	"
26	AG1	JF701755	RMHG24	"	India	"
27	AG1	JF701751	RMPG29	"	India	"
28	AG2-3	JF701750	RMPG28	"	India	"
29	AG5	JF701722	RGJG2	"	India	"
30	AG3	JF701718	RDLG3	"	India	"
31	AG2-3	JF701708	RAPG14	"	India	"
32	AG4	EU730836	R176	Root rot	Canada	[50]
33	AG10	DQ356408	Rh100278	Root rot	USA	[38]

Table S4. Data regarding anastomosis groups of *R. solani* associated with common beans.

S. No	Anastomosis Group	Accession numbers	Isolates	Pathogenicity	Geo-graphic origin	References
1	AG2-2 IIIB	FJ492171.3	F561	Root rot	USA	[74]
2	AG2-2 IIIB	FJ492170.3	F560	Root rot	USA	"
3	AG1-IE	AF308629.2	PR5	Web blight	Puerto Rico	[15]
4	AG1-IF	AF308627.2	H32	"	Honduras	"
5	AG1-IF	AF308625.2	DR-BV1	"	Dominican Republic	"
6	AG2-2 WB	AF308621.2	A4	"	Argentina	"
7	AG2-2 WB	DQ452119	Barranca	"	Dominican Republic	"
8	AG2-2 WB	DQ452115	AL2002	"	Dominican Republic	"
9	AG2-2 WB	DQ452111	EB3	"	Ecuado	"
10	AG2-2 WB	DQ452110	DR-LV-1	"	Dominican Republic	"
11	AG2-2 WB	DQ452109	Los_Bancos	"	Dominican Republic	"
12	AG2-2 WB	DQ452108	H43	"	Honduras	"
13	AG2-2 WB	DQ452104	H2002-1	"	Honduras	"
14	AG2-2 WB	DQ452102	E8-1	"	Ecuador	"
15	AG2-2 WB	DQ452101	E3-3	"	Ecuador	"
16	AG2-2 WB	DQ452100	E2-1	"	Ecuador	"
17	AG2-2 WB	DQ452099	E1-1	"	Ecuador	"
18	AG2-2 WB	DQ452098	MGB1	"	Honduras	"
19	AG4-HGII	HE805667	M47ZD	Seed and root rot, damping off	Turkey	[41]
20	AG4-HGI	HE805691	M91KK	"	Turkey	"
21	AG4-HGII	HE805690	M47ZD	"	Turkey	"
22	AG4-HGI	HE805689	M46ZA	"	Turkey	"
23	AG4-HGI	HE805688	M72GP	"	Turkey	"
24	AG4-HGI	HE805687	M54ZG	"	Turkey	"
25	AG4-HGI	HE805686	M67ZA	"	Turkey	"
26	AG4-HGI	HE805685	M88GE	"	Turkey	"
27	AG4-HGI	HE805684	M89GE	"	Turkey	"
28	AG4-HGI	HE805683	M75GP	"	Turkey	"
29	AG4-HGI	HE805682	M63ZC	"	Turkey	"
30	AG4-HGI	HE805681	M93KD	"	Turkey	"
31	AG4-HGI	HE805680	M86GD	"	Turkey	"
32	AG4-HGI	HE805679	M76GK	"	Turkey	"
33	AG4-HGI	HE805678	M51KM	"	Turkey	"
34	AG4-HGI	HE805677	M52GD	"	Turkey	"

35	AG4-HGI	HE805676	M69ZC	"	Turkey	"
36	AG4-HGI	HE805675	M64ZG	"	Turkey	"
37	AG4-HGI	HE805674	M74GK	"	Turkey	"
38	AG4-HGI	HE805673	M56KD	"	Turkey	"
39	AG4-HGI	HE805672	M70ZG	"	Turkey	"
40	AG4-HGI	HE805671	M62ZG	"	Turkey	"
41	AG4-HGI	HE805670	M57KM	"	Turkey	"
42	AG4-HGII	HE805669	M60SA	"	Turkey	"
43	AG4-HGII	HE805668	M48KP	"	Turkey	"
44	AG4-HGI	HE805666	M91KK	"	Turkey	"
45	AG2-2WB	KU361244	Rs98B	Web blight	Argentina	[23]
46	AG2-2WB	KU361245	Rs99B	"	Argentina	"
47	AG2-2WB	KU361246	Rs100B	"	Argentina	"
48	AG2-2WB	KU361247	Rs106B	"	Argentina	"
49	AG2-2WB	KU361248	Rs107B	"	Argentina	"
50	AG2-2WB	KU361249	Rs108B	"	Argentina	"
51	AG2-2WB	KU361250	Rs109B	"	Argentina	"
52	AG2-2WB	KU361251	Rs110B	"	Argentina	"
53	AG2-2WB	KU361252	Rs112B	"	Argentina	"
54	AG2-2WB	KU361253	Rs113B	"	Argentina	"
55	AG2-2WB	KU361254	Rs114B	"	Argentina	"
56	AG2-2WB	KU361255	Rs115B	"	Argentina	"
57	AG2-2WB	KU361256	Rs117B	"	Argentina	"
58	AG2-2WB	KU361257	Rs118B	"	Argentina	"
59	AG2-2WB	KU361258	Rs119B	"	Argentina	"
60	AG2-2WB	KU361259	Rs121B	"	Argentina	"
61	AG2-2WB	KU361260	Rs122B	"	Argentina	"
62	AG2-2WB	KU361261	Rs130B	"	Argentina	"
63	AG2-2WB	KU361262	Rs131B	"	Argentina	"
64	AG2-2WB	KU361263	Rs133B	"	Argentina	"
65	AG2-2WB	KU361264	Rs134B	"	Argentina	"
66	AG2-2WB	KU361265	Rs136B	"	Argentina	"
67	AG2-2WB	KU361266	Rs137B	"	Argentina	"
68	AG2-2WB	KU361267	Rs139B	"	Argentina	"
69	AG2-2WB	KU361268	Rs141B	"	Argentina	"
70	AG2-2WB	KU361269	Rs144B	"	Argentina	"
71	AG2-2WB	KU361270	Rs146B	"	Argentina	"
72	AG2-2WB	KU361271	Rs147B	"	Argentina	"
73	AG2-2WB	KU361272	Rs148B	"	Argentina	"
74	AG2-2WB	KU361273	Rs151B	"	Argentina	"
75	AG2-2WB	KU361274	Rs153B	"	Argentina	"
76	AG2-2WB	KU361275	Rs154B	"	Argentina	"
77	AG2-2WB	KU361276	Rs159B	"	Argentina	"
78	AG2-2WB	KU361277	Rs160B	"	Argentina	"
79	AG2-2WB	KU361278	Rs167B	"	Argentina	"
80	AG2-2WB	KU361279	Rs169B	"	Argentina	"
81	AG2-2WB	KU361280	Rs170B	"	Argentina	"

82	AG2-2WB	KU361281	Rs171B	"	Argentina	"
83	AG2-2WB	KU361282	Rs172B	"	Argentina	"
84	AG2-2WB	KU361283	Rs174B	"	Argentina	"
85	AG2-2WB	KU361284	Rs175B	"	Argentina	"
86	AG2-2WB	KU361285	Rs176B	"	Argentina	"
87	AG2-2WB	KU361286	Rs177B	"	Argentina	"
88	AG2-2WB	KU361287	Rs178B	"	Argentina	"
89	AG2-2WB	KU361288	Rs180B	"	Argentina	"
90	AG2-2WB	KU361289	Rs183B	"	Argentina	"
91	AG2-2WB	KU361290	Rs295/1B	"	Argentina	"
92	AG2-2WB	KU361291	Rs295/2B	"	Argentina	"
93	AG2-2WB	KU361292	Rs295/3B	"	Argentina	"
94	AG2-2WB	KU361293	Rs295/4B	"	Argentina	"

Table S5. Data regarding anastomosis groups of *R. solani* associated with pea.

Iso-lates	Anastomosis Group	Accession number	Isolates	Patho-genicity	Geographic origin	References
1	AG-4	EU730828	R93	Seedling blight and root rot	Canada	[50]
2	AG-4	EU730827	R92	"	Canada	"
3	AG-4	EU730821	R73	"	Canada	"
4	AG-4	EU730820	R71	"	Canada	"
5	AG2-2	JF701756	RMHP21	Web/fo-liar blight/wet root rot	India	[14]
6	AG-4	JF701752	RMPP30	"	India	"
7	AG4-HGII	HQ629876	ND17	Root and stem rot	USA	[40]
8	AG4-HGII	HQ629875	ND14	"	USA	"
9	AG5	HQ629874	ND2	"	USA	"
10	AG4-HGII	HQ629873	ND13	"	USA	"
11	AG4-HGII	HQ629872	ND12	"	USA	"
12	AG4-HGII	HQ629871	ND11	"	USA	"
13	AG4-HGII	HQ629870	ND10	"	USA	"
14	AG4-HGII	HQ629869	ND9	"	USA	"
15	AG4-HGII	HQ629868	ND8	"	USA	"
16	AG4-HGII	HQ629867	ND7	"	USA	"
17	AG4-HGII	HQ629866	ND6	"	USA	"
18	AG4-HGII	HQ629865	ND16	"	USA	"
19	AG4-HGII	HQ629864	ND15	"	USA	"
20	AG4-HGII	HQ629862	ND5	"	USA	"
21	AG4-HGII	HQ629861	ND4	"	USA	"
22	AG4-HGII	HQ629860	ND3	"	USA	"
23	AG4-HGII	HQ629859	ND2	"	USA	"

24	AG4-HGII	HQ629858	ND1	"	USA	"
25	AG2-1	KF870891	10-D4_16R	Root and stem rot, damping off	Canada	[75]
26	AG2-1	U57722	021R01	None	Japan	[21]

Table S6. Data regarding anastomosis groups of *R. solani* associated with soybean.

Isolates	Anastomosis Group	Accession numbers	Isolates	Pathogenic-ity	Geographic origin	References
1	AG1-IA	AF354060	1Rs		USA	[22]
2	AG5	AF354078	10Rs		Japan	"
3	AG5	KX118386	USA_AG-5	Damping off, root and hypocotyl rot	USA	[3]
4	AG3	KX118385	USA_AG-3	"	USA	"
5	AG7	KX118384	ST81548	"	USA	"
6	AG11	KX118382	SP_19b	"	USA	"
7	AG11	KX118381	SP_19a	"	USA	"
8	AG5	KX118377	Rh0911029	"	USA	"
9	AG2-1	KX118376	Rh051324	"	USA	"
10	AG2-1	KX118375	Rh051307	"	USA	"
11	AG2-2IIIB	KX118373	PDONS_13_8_1	"	Canada	"
12	AG2-2IIIB	KX118372	ONS02_18	"	Canada	"
13	AG11	KX118367	K_4_18b	"	USA	"
14	AG2-1	KX118363	KARS02_5_1	"	USA	"
15	AG7	KX118362	KARS02_2_5	"	USA	"
16	AG7	KX118361	KARS02_1_9	"	USA	"
17	AG7	KX118360	KARS02_1_8	"	USA	"
18	AG7	KX118359	KARS02_1_6	"	USA	"
19	AG11	KX118358	KARS02_1_20	"	USA	"
20	AG11	KX118356	KARS02_1_11	"	USA	"
21	AG11	KX118355	HPIN22A	"	USA	"
22	AG7	KX118354	EV_7	"	USA	"
23	AG4-HGI	KX118353	EV_6	"	USA	"
24	AG4-HGI	KX118352	EV_3	"	USA	"
25	AG7	KX118351	EV_19	"	USA	"
26	AG2-2IIIB	KX118350	ER_4	"	USA	"
27	AG2-2IIIB	KX118349	ER_2	"	USA	"
28	AG2-2IIIB	KX118348	ER_19b	"	USA	"
29	AG2-2IIIB	KX118347	ER_19a	"	USA	"
30	AG2-2IIIB	KX118346	ER_15	"	USA	"
31	AG2-2IIIB	KX118345	DK_8	"	USA	"
32	AG2-2IIIB	KX118344	DK_11	"	USA	"

33	AG3	KX118343	Cfar_500_6	"	USA	"
34	AG3	KX118342	Cfar_500_3	"	USA	"
35	AG3	KX118341	Cfar_500_10a	"	USA	"
36	AG3	KX118340	Cfar_500_1	"	USA	"
37	AG4-HGIII	KX118339	BVT_3	"	USA	"
38	AG4-HGIII	KX118338	BVT_28	"	USA	"
39	AG7	KX118337	BVT_20	"	USA	"
40	AG11	KX118336	BVT_18	"	USA	"
41	AG7	KX118335	BVT_16	"	USA	"
42	AG2-2IIIB	KX118334	65L-2	"	USA	"
43	AG4-HGIII	KX118331	AG-4_Carling	"	USA	"
44	AG4-HGIII	KX118330	AG-4_Nelson	"	USA	"
45	AG4-HGIII	KX118329	Rh051339	"	USA	"
46	AG2-2	KX118328	AG2-2_Nel-son	"	USA	"
47	AG2-2-IIIB	KX118397	X42210_b	"	USA	"
48	AG3	KX118396	X248_3bKH	"	USA	"
49	AG11	KX118394	X12SDSa	"	USA	"
50	AG2-2IIIB	KX118392	X12Rs41	"	USA	"
51	AG2-2IIIB	KX118391	WONS_13_8_5	"	USA	"
52	AG2-2IIIB	KX118390	WONS_13_12_3	"	USA	"
53	AG-1	JF701709	RAPS3	Web/foliar blight/wet rot	India	[14]
54	AG2-3	FJ435110	CSL1934	Damping off	Japan	[54]
55	AG2-3	FJ435099	CSL1866	Damping off	Japan	"
56	AG7	AF153793	Roth32	none	USA	[76]
57	AG11	AF354115	Roth24		USA	[20]
58	AG11	AF354114	Roth16		USA	"
59	AG5	AF354112	19Rs		Japan	"
60	AG4	AF354081	BN38		USA	"
61	AG1-IA	DQ173075	SJ142-2	Leaf blight	Brazil	[30]
62	AG1-IA	DQ173074	SJ140	"	Brazil	"
63	AG1-IA	DQ173073	SJ134	"	Brazil	"
64	AG1-IA	DQ173072	SJ133	"	Brazil	"
65	AG1-IA	DQ173071	SJ129	"	Brazil	"
66	AG1-IA	DQ173070	SJ127	"	Brazil	"
67	AG1-IA	DQ173069	SJ121	"	Brazil	"
68	AG1-IA	DQ173068	SJ093	"	Brazil	"
69	AG1-IA	DQ173067	SJ080	"	Brazil	"
70	AG1-IA	DQ173066	SJ064	"	Brazil	"
71	AG1-IA	DQ173065	SJ053	"	Brazil	"
72	AG1-IA	DQ173064	SJ048	"	Brazil	"

73	AG1-IA	DQ173063	SJ047	"	Brazil	"
74	AG1-IA	DQ173062	SJ044	"	Brazil	"
75	AG1-IA	DQ173061	SJ040	"	Brazil	"
76	AG1-IA	DQ173060	SJ036	"	Brazil	"
77	AG1-IA	DQ173059	SJ031-6	"	Brazil	"
78	AG1-IA	DQ173058	SJ031-5	"	Brazil	"
79	AG1-IA	DQ173057	SJ023	"	Brazil	"
80	AG1-IA	DQ173056	SJ021	"	Brazil	"
81	AG1-IA	DQ173055	SJ015	"	Brazil	"
82	AG1-IA	DQ173054	SJ014-2	"	Brazil	"
83	AG1-IA	DQ173053	SJ013	"	Brazil	"
84	AG1-IC	DQ173077	AG-1 IC	"	USA	"
85	AG1-IB	DQ173076	AG-1IB	"	USA	"
86	AG1-IA	DQ173052	Rhs-9F1	"	Brazil	"
87	AG1-IA	DQ173051	Rhs-4F1	"	Brazil	"
88	AG1-IA	DQ173050	Rhs-3F6	"	Brazil	"
89	AG1-IA	DQ173049	Rhs-3F1	"	Brazil	"
90	AG1-IA	DQ173048	AG1IArJ	"	Japan	"
91	AG1-IA	DQ173047	AG1IAmJ	"	Japan	"
92	AG4-HGII	AY270005	Not available	Hypocotyle rot	Brazil	[28]
93	AG2-2IIIB	AY270015	SJ07		Brazil	[20]
94	AG1-IA	AY270013	SJ19	Foliar blight	Brazil	[28]
95	AG1-IA	AY270012	SJ57	Foliar blight	Brazil	"
96	AG1-IA	AY270011	SJ67	Foliar blight	Brazil	"
97	AG1-IA	AY270010	SJ16	Foliar blight	Brazil	"
98	AG1-IA	AY270009	SJ26	Foliar blight	Brazil	"
99	AG1-IA	AY270008	SJ24	Foliar blight	Brazil	"
100	AG1-IA	AY270007	SJ34	Foliar blight	Brazil	"
101	AG1-IA	AY270006	SJ28	Foliar blight	Brazil	"
102	AG4-HGII	AY270004	SJ03	Hypocotyle rot	Brazil	"
103	AG4-HGII	AY270003	SJ02	Hypocotyle rot	Brazil	"
104	AG4-HGII	AY270002	SJ05	Hypocotyle rot	Brazil	"
105	AG4-HGII	AY270001	SJ01	Hypocotyle rot	Brazil	"
106	AG9	KX118333	unk	none	USA	[47]
107	AG6	KX118332	AG-6_Carling	none	none	

Table S7. Best substitution models used in this study.

Best model from jModel test	Abbreviations	Substitution Models	Rates among sites
**Common bean**			
AIC	TrN+I+G	Tamura-Nei (TrN)	Gamma distributed with invariant sites
BIC	TrN+G	Tamura-Nei (TrN)	Gamma distributed
AICc	JC+G	Jukes-Cantor	Gamma distributed
DT	TVM+I	Transversion model	Invariant sites
**Cowpea and peanuts**			
AIC	TIM1+G	Transversion model	Gamma distributed
BIC	HKY+G	Hasegawa-Kishino-Yano	Gamma distributed
AICc	TIM1+G	transversion model	Gamma distributed
DT	TIM1+G	transversion model	Gamma distributed
**Soybean**	**Models**		
AIC	TIM1+I+G	transversion model	Gamma distributed with invariant sites
BIC	TrN+G	Tamura-Nei (TrN)	Gamma distributed
AICc	TrN+G	Tamura-Nei (TrN)	Gamma distributed
DT	TrN+G	Tamura-Nei (TrN)	Gamma distributed
**Chickpea**			Gamma distributed
AIC	TrN+G	Tamura-Nei (TrN)	Gamma distributed
BIC	TrN+G	Tamura-Nei (TrN)	Gamma distributed
AICc	TrN+G	Tamura-Nei (TrN)	Gamma distributed
DT	HKY+G	Hasegawa-Kishino-Yano	Gamma distributed
**Pea**	**Models**		
AIC	TrN+G	Tamura-Nei (TrN)	Gamma distributed
BIC	HKY+G	Hasegawa-Kishino-Yano	Gamma distributed
AICc	TrN+G	Tamura-Nei (TrN)	Gamma distributed
DT	TrN+G	Tamura-Nei (TrN)	Gamma distributed
**Alfalfa and clovers**			
AIC	TPM3uf+G (GTR)	General time-reversible (GTR)	Gamma distributed
AIC	TPM3uf+G (GTR)	General time-reversible (GTR)	Gamma distributed
AIC	TPM3uf+G (GTR)	General time-reversible (GTR)	Gamma distributed
AIC	TPM3uf+G (GTR)	General time-reversible (GTR)	Gamma distributed
**Best models from MEGA-X**			
**Cowpea and peanuts**			
BIC	T92	Timura 3-parameter	None
AICc	T93+G	Tamura-Nei (TrN)	Gamma distributed
**Chickpea**			
BIC	T92+I	Timura 3-parameter	Invariant sites
AICc	T93+G+I	Tamura-Nei (TrN)	Gamma distributed with invariant sites

**Common bean**			
BIC	T93+G	Timura 3-parameter	Gamma distributed
AICc	T92+G+I	Tamura-Nei (TrN)	Gamma distributed with invariant sites
**Soybean**			
BIC	T92+G	Timura 3-parameter	Gamma distributed
AICc	T93+G+I	Tamura-Nei (TrN)	Gamma distributed with invariant sites
**Alfalfa and clover**			
BIC	T92	Timura 3-parameter	None
AICc	T92+G	Timura 3-parameter	Gamma distributed
**Pea**			
BIC	T92+I	Timura 3-parameter	Invariant sites
AICc	T93+G	Tamura-Nei (TrN)	Gamma distributed
**All legumes**	T93+G	Tamura-Nei (TrN)	Gamma distributed
	T92+G	Timura 3-parameter	Gamma distributed

Table S8. Number of anastomosis groups (AGs) sequences of *R. solani* associated with legumes crop.

Anastomosis groups		Legume crops									
**[a]AGs**	**[b]Sub.**	**Alfalfa**	**Clover**	**Soybean**	**Common bean**	**Chickpea**	**Pea**	**Peanut**	**Cowpea**	**Total**	
AG-1	1			1		2				3	
	IA	1		38				1	1	41	
	IB		6	1						7	55
	IC			1						1	
	IE				1					1	
	IF				2					2	
AG-2	1			3			2			5	
	2			1			1			2	
	2IIIB			18	2					20	94
	2LP									0	
	2WB				63					63	
	3			2		2				4	
AG-3	3			6		2		1		9	9
AG-4	4			1		1	5	1		8	
	HGI			2	22			2		26	87
	HGII	2		4	4	23	17			50	
	HGIII			3						3	
AG-5	[c]NA			4		2	1			7	7
AG-6	[c]NA			0						0	0
AG-7	[c]NA			10						10	10
AG-9	[c]NA			1						1	1
AG-10	[c]NA					1				1	1
AG-11	[c]NA		1	10						11	11
Unc	[c]NA								2	2	2
Total		3	7	107	94	33	26	5	3	277	

[a]AGs = Anastomosis groups, [b]Sub. = AGs subgroup, [c]NA = Not applicable/available, Unc = Unclassified.

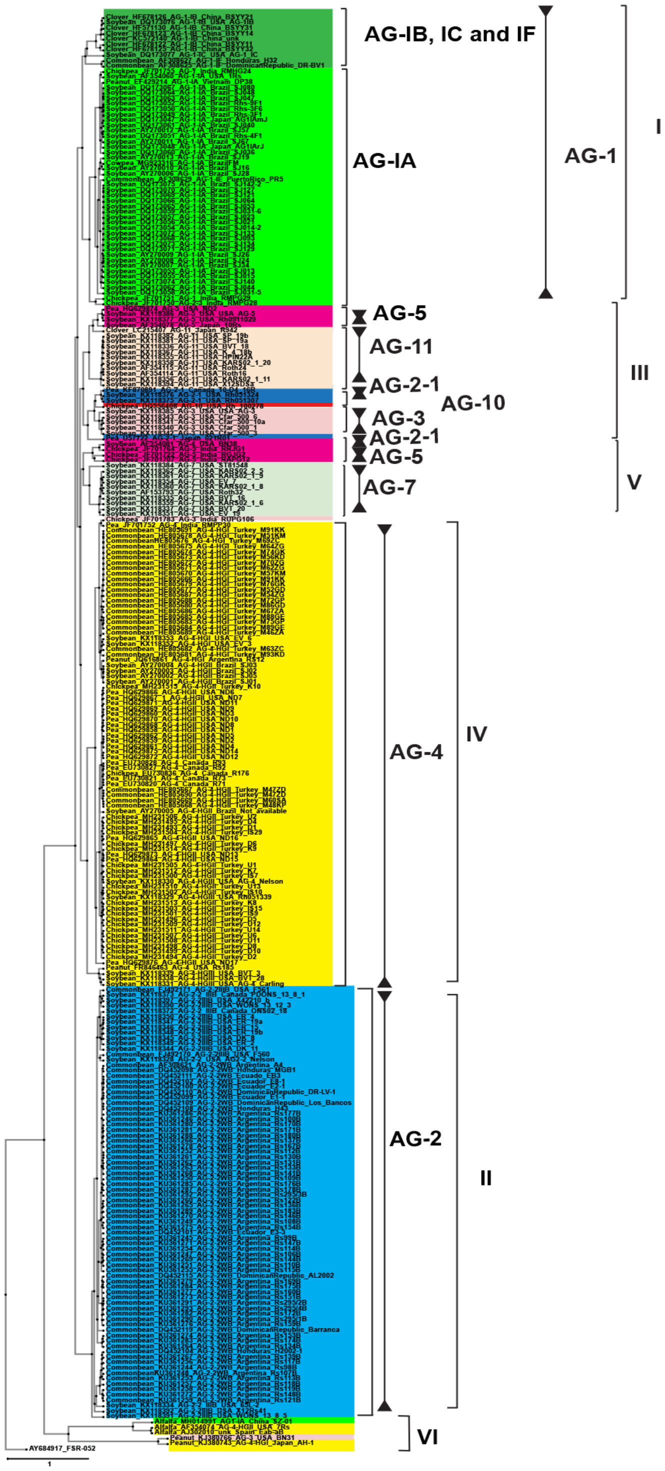

Figure S1. Phylogenetic trees of AG sequences associated with all legume crops using the Neighbor-Joining (NJ) method. The common name of the legume crop is followed by reference accession numbers, AGs from the GenBank, geographical origin, and isolate name. Different colors show AGs and clades and/or subclades associated with AGs. Roman numerals (I-VI) represent groups or clusters or clades. **a.** Neighbor-Joining (NJ) tree; *Athelia rolfsii* (strain FSR-052) was used as an outgroup.
